# Electric Signals Regulate the Directional Migration of Oligodendrocyte Progenitor Cells (OPCs) via β1 Integrin

**DOI:** 10.3390/ijms17111948

**Published:** 2016-11-22

**Authors:** Bangfu Zhu, Matthew Nicholls, Yu Gu, Gaofeng Zhang, Chao Zhao, Robin J. M. Franklin, Bing Song

**Affiliations:** 1 Cardiff Institute of Tissue Engineering and Repair, School of Dentistry, College of Biomedical and Life Sciences, Cardiff University, Cardiff CF14 4XY, UK; nsxbz@bristol.ac.uk (B.Z.); nichollsm2@cardiff.ac.uk (M.N.); guY11@cardiff.ac.uk (Y.G.); gaofengzhang@gmail.com (G.Z.); 2 School of Biochemistry, University of Bristol, Bristol BS8 1TD, UK; 3 Wellcome Trust—Medical Research Council Cambridge Stem Cell Institute, University of Cambridge, Cambridge CB2 1TA, UK; cz213@cam.ac.uk (C.Z.); rjf1000@cam.ac.uk (R.J.M.F.); 4 Department of Dermatology, No. 1 Hospital of China Medical University, Shenyang 110001, China

**Keywords:** electric field, oligodendrocyte progenitor, cell migration, integrin

## Abstract

The guided migration of neural cells is essential for repair in the central nervous system (CNS). Oligodendrocyte progenitor cells (OPCs) will normally migrate towards an injury site to re-sheath demyelinated axons; however the mechanisms underlying this process are not well understood. Endogenous electric fields (EFs) are known to influence cell migration in vivo, and have been utilised in this study to direct the migration of OPCs isolated from neonatal Sprague-Dawley rats. The OPCs were exposed to physiological levels of electrical stimulation, and displayed a marked electrotactic response that was dependent on β1 integrin, one of the key subunits of integrin receptors. We also observed that F-actin, an important component of the cytoskeleton, was re-distributed towards the leading edge of the migrating cells, and that this asymmetric rearrangement was associated with β1 integrin function.

## 1. Introduction

Oligodendrocytes are the myelinating cells of the central nervous system (CNS), and are essential for both the high-speed conduction of action potentials [1] and the maintenance of neuronal circuitry [2]. The majority of oligodendrocytes develop during embryogenesis and, along with their oligodendrocyte progenitor cell (OPC) precursors, originate from both the ventral ventricular zone of the embryonic spinal cord and the subventricular region of the embryonic brain [3,4,5]. OPCs typically display a bipolar/tripolar morphology, and can be identified by their expression of various antigens [6]; namely the NG2 chondroitin sulfate proteoglycan, the platelet-derived growth factor-alpha receptor (PDGF-αR) [7], and the membrane ganglioside A2B5. Although the principal physiological function of OPCs is to differentiate into myelinating oligodendrocytes during development, these cells are able to facilitate the regenerative process by remyelinating naked axons following injury [8,9]. These qualities identify OPCs as a promising cellular platform upon which to base a therapeutic approach for dysmyelinating/demyelinating diseases. Genetic diseases such as familial leukodystrophies [10], or comparatively prevalent conditions such as spinal cord injury (SCI) or multiple sclerosis (MS), are likely to benefit from an OPC-based therapy that aims to remyelinate axons and restore peak neuronal function [11,12,13].

DC (direct current) voltage gradients exist naturally within organic tissues and represent a facet of regeneration that, until recently, has not often been studied. For example, a transepithelial potential is maintained across the epithelial layers of human skin and, upon laceration, a localized electric field occurs and persists as current leaks through the wound. In an electrical circuit, current is carried by the flow of electrons along electroconductive wires. In a physiological setting, ions carry electric current through a conductive medium; such as the cell cytoplasm or extracellular fluids. A voltage difference between two loci within such a medium will generate a current. The current per unit area (*J*) and the resistivity of the conductive medium (ρ) form a relationship with the electric field (*E*), such that *E* = *J*ρ. Both *E* and *J* are vectors and exhibit both magnitude and direction, thus electric fields can induce the directional migration of chemicals, molecules and cells [14]. To put the magnitude of these electrical signals into context; depolarizing a neuron and initiating an action potential using surface electrodes requires 1–2 V/mm of electric field stimulation. Conventional electroporation methods used to perform gene transfection or facilitate drug delivery into target cells will utilize relatively large pulses of DC signals that normally fall between 100 and 500 V/mm. DC electric fields (EFs) that occur during development or as part of the natural regenerative process are far lower in magnitude, and fall between 1–100 mV/mm [14].

Endogenous EFs play a significant role in the development of the central nervous system, and are capable of regulating cell migration [14]. Given the role EFs play in neurogenesis, nerve growth, and axon guidance [14,15], it can be reasoned that EFs of a physiological magnitude might act as a potential guidance cue to regulate the guided migration of OPCs [16]. If grafted OPCs could be successfully directed to a target injury site, they may promote the functional remyelination of demyelinated axons. Unfortunately, the mechanism for regulating the directional migration of OPCs is not yet fully understood. One of the more extensively examined responses of neural cells in an EF concerns the cathodal orientation of the neuronal growth cone [14]. Initially, physiological EFs will induce the physical movement of charged receptor molecules exposed on the lipid bilayer. This forces an asymmetric cathodal distribution of receptors relative to the anode-oriented pole of the growth cone. The pertinent membrane receptors that respond to EF stimulation vary considerably between different cell types. Acetylcholine receptors (AChRs) are the putative receptors concerning the neuronal growth cone response to electrical stimulation: given their tendency to orient cathodally when exposed to an EF, to spontaneously secrete acetylcholine (ACh), and to actively orientate towards sources of ACh. As a consequence of their mechanics, operational AChRs will cause levels of intracellular Ca^2+^ to rise as extracellular Ca^2+^ ions leak through the active receptors. Mandatory activation of the receptors trkB and trkC by their respective ligands, BDNF and NT-3, causes an increase in ACh secretion and further polarizes AChR distribution. Levels of intracellular Ca^2+^ are increased yet further as AChRs and trkB receptors activate the phospholipase-C (PLC) and phosphatidylinositol 3-kinase (PI3K) pathways. This net Ca^2+^ elevation stimulates cAMP production and activates protein kinase A (PKA), which in turn activates the small GTPases rac1, rhoA and cdc42. The activation of GTPases rac1 and cdc42 is thought to underlie both the formation and the EF-induced orientation of lamellipodia and filopodia situated on the cathodal face of the neuronal growth cone. The inhibition of rhoA by PKA will prevent cathodal growth cone collapse, but will lead to anodal growth cone collapse; thus generating an asymmetric tension within the grown cone to result in cathodal orientation. Consequently, the elevation of intracellular Ca^2+^ is critical to growth cone orientation [14].

Existing literature indicates that OPCs, which tend to migrate over greater distances than other types of neural cell, exhibit a motility that is similarly determined by various internal mechanisms and extracellular signals [17]. The gene encoding myelin basic protein (MBP) also codes for the family of golli proteins, which are expressed in both neurons and oligodendrocytes when they extend processes for migration [18]. It was found that disruption to golli expression in oligodendrocytes leads to defective myelin production, whereas overexpression of golli appears to improve the quality of myelin sheets and the extension of migration processes [18]. These improvements were negated, however, when voltage-gated Ca^2+^ channels (VGCCs) were blocked using a specific VGCC blocker—Cd^2+^—which suggests that golli-mediated enhancements to process extension require a sufficient influx of Ca^2+^. Further analysis of OPC migratory processes revealed that areas with higher levels of golli expression were associated with stronger influxes of Ca^2+^ [18]. Given that process extension and retraction plays a critical role in cell migration, it seemed likely that golli would influence OPC motility. Following an investigation, Paez et al. (2009) [18] showed that OPCs with increased golli expression were more motile, and were accompanied by relative increases of subcellular Ca^2+^ uptake. In vivo, the migratory progenitor cell type is believed to belong to the oligodendrocyte-type 2 astrocyte (O-2A) lineage; a cell from which is identifiable by its expression of A2B5 [9]. A2B5-positive OPCs have been previously demonstrated to migrate significant distances and construct myelin after transplantation into hypomyelinating *Shiverer* mice [19]. In this study, we show that primary, A2B5-positive rat OPCs will undergo electrotaxis when exposed to a physiological-strength EF.

We have previously demonstrated that electrical stimulation will direct the migration of grafted neural stem cells via the PI3K/Akt signaling pathway [20]—which has already been widely explored for its role in the cellular response to electrical stimulation; including its role in the migration, proliferation and differentiation of numerous cell types [20]. However, several signaling pathways are now known to play a role in the cellular response to electrical stimulation [21]. Integrin signaling pathways are crucial for the survival and maturation of oligodendrocytes, and play a part in triggering the downstream signaling pathways that are responsible for cytoskeletal remodeling [4]. A recent study has shown that OPC migration entails a dynamic cytoskeletal rearrangement [22], which is likely to be at least partially dependent upon integrin signaling. This led us to examine the link between integrin signaling and the cytoskeletal remodeling that occurs during OPC migration.

In this study, we explore the use of EFs, set at a physiological magnitude, to regulate the directed migration of OPCs derived from the brains of neonatal Sprague-Dawley rats. We demonstrate that OPCs show marked electrotaxis, and that their directional migration is dependent on one of the key subunits of integrin receptors: β1 integrin. We also show that F-actin, a key component of the cytoskeleton, is re-distributed towards the leading edge of migrating OPCs.

## 2. Results

### 2.1. Identification of Rat Oligodendrocyte Progenitor Cells (OPCs)

**Figure 1:**
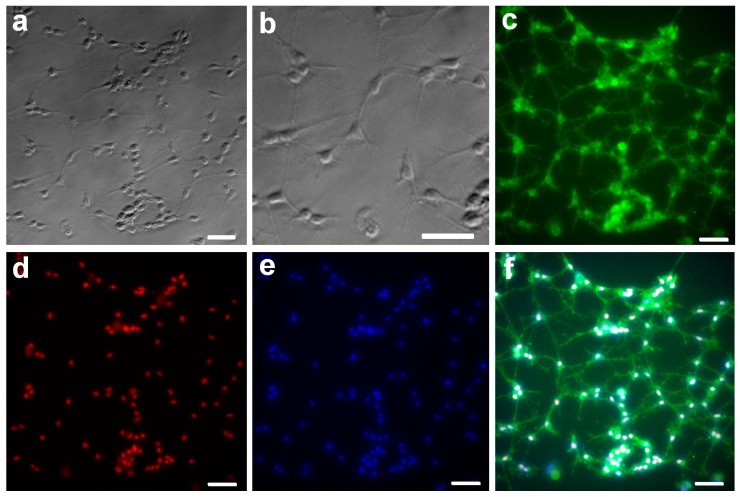
Identification of isolated rat oligodendrocyte progenitor cells (OPCs). OPCs typically display a bipolar/tripolar morphology, with little evidence of secondary branching 3 days after isolation (**a**). Higher magnification of (**a**) allows smaller, developing processes to be picked out (**b**). OPCs are characterized as immunopositive for A2B5 (**c**, **green**) and Olig2 (**d**, **red**). Nuclear counter-staining of OPCs with DAPI (**e**, **blue**). The DAPI channel is combined with the A2B5 and Olig2 channels (**f**). Scale bars = 50 μm.

We have previously published a protocol for the isolation, expansion and long-term culture of rat OPCs, which delivers the cells in highly purified populations [23]. Proliferating OPCs can be identified by their expression of specific progenitor cell markers, such as the platelet-derived growth factor-alpha receptor (PDGF-αR) or the membrane ganglioside A2B5. Following separation from mixed-glial cultures, the isolated OPCs grew rapidly in PDGF/bFGF-supplemented culture medium, and initially displayed a typically bipolar/tripolar morphology; with little or no evidence of secondary branching (Figure 1a,b). In order to characterize the properties of the isolated cells, we identified the cells as OPCs through immunocytochemical (ICC) staining. The immunostaining results revealed that the majority of isolated cells expressed the OPC-specific marker A2B5 (Figure 1c). All cells were positive for the OPC lineage nuclear marker Olig2 (Figure 1d) and the nuclear marker DAPI (Figure 1e).

### 2.2. Characterization of Differentiated OPCs

The growth factors PDGF and bFGF will promote OPC division, and are conducive to the maintenance of an OPC population. Conversely, T3/T4 treatment will prohibit OPC proliferation to induce their differentiation into oligodendrocytes. T3 also advances the morphological maturation of post-mitotic oligodendrocytes. Therefore, the withdrawal of PDGF and bFGF, followed by T3/T4 treatment, will result in OPC differentiation [23]. We also noticed that a low concentration (0.5%) of FBS will further promote the differentiation process. Oligodendrocyte differentiation from progenitor cells is characterized by significant changes to cellular morphology and antigenicity. As OPCs differentiate and mature, additional processes develop and extend from the cell body to form secondary and tertiary branches (Figure S1a). Changes in the expression of cell-specific markers can also be used to follow the differentiation process. OPCs grown as single cells under differentiation conditions will eventually mature into myelin-producing oligodendrocytes, which can be characterized by their exclusive expression of MBP (Figure S1b). All cells remained positive for Olig2 (Figure S1c).

### 2.3. Response of OPCs to Electric Field (EF) Stimulation

**Figure 2:**
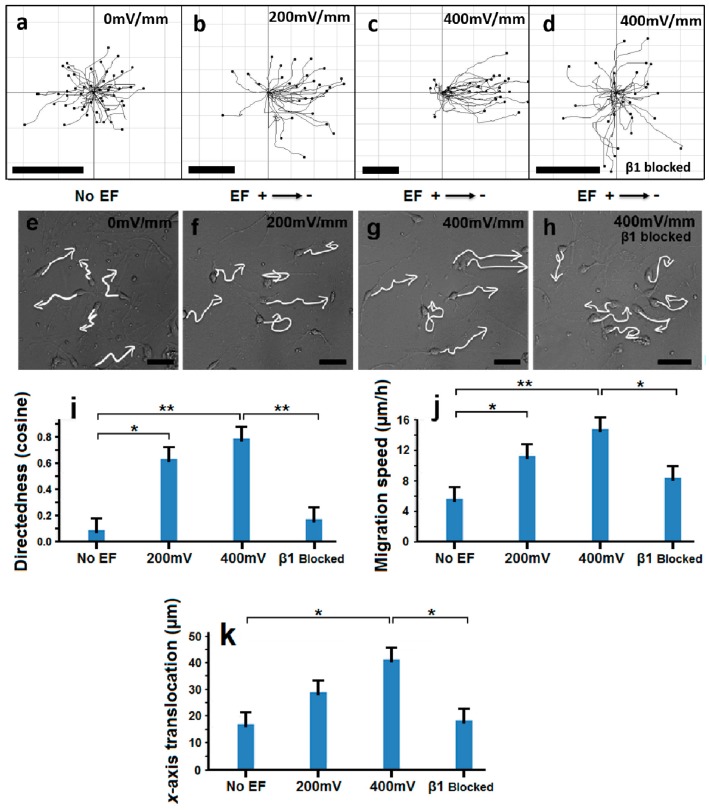
Electric field (EF)-directed OPC migration is dependent on β1 integrin. Recorded migration trajectories of OPCs over a 4 h period under different treatment conditions: no EF (**a**); 200 mV/mm (**b**); 400 mV/mm (**c**); and 400 mV/mm with β1 integrin inhibition (**d**). The migration trajectories of OPCs over a 4 h period are described by the white lines and arrows: no EF (**e**); 200 mV/mm (**f**); 400 mV/mm (**g**); and 400 mV/mm with β1 integrin inhibition (**h**). The value of the migration directedness (the average cosine of all angles measured between the path of cell migration and the *x*-axis) represents the net OPC translocation towards the cathode measured over a 4 h period. OPCs that were exposed to 200 and 400 mV/mm EFs displayed a significantly greater directedness compared to OPCs that were either not EF-treated, or were treated to a 400 mV/mm EF with β1 integrin inhibition (**i**). A similar pattern is observed regarding OPC migration speed (**j**). OPCs exposed to a 400 mV/mm EF displayed greater *x*-axis translocation towards the cathode when compared to OPCs that were either not EF-treated, or were treated to a 400 mV/mm EF with β1 integrin inhibition (**k**). The starting points of the all recorded migration trajectories were set at the origin of the *x*-*y* axes (0, 0). Refer to Supplemental Videos 1–4. (* *p* < 0.05; ** *p* < 0.01). Scale bar = 50 μm.

We have previously demonstrated that neural stem/progenitor cells will directionally migrate in response to electrical stimulation [20]. Here we investigate whether or not OPCs isolated from neonatal rats will respond to the electrical signals generated by an electric field (EF). We initially observed that, in the absence of electrical stimulation, the majority (about two thirds) of OPCs displayed little or no movement. Those cells that did move were found to migrate in random directions (Figure 2a,e; Supplemental Video 1). When exposed to a 200 mV/mm EF, most OPCs moved slowly and in a similar direction (Figure 2b,f; Supplemental Video 2). When the EF strength was increased to 400 mV/mm, the OPCs moved over a significantly greater distance (Figure 2c,g; Supplemental Video 3). Directedness analysis showed that the majority of cells moved towards the cathode of the EF (Figure 2i; Supplemental Videos 2 and 3). Furthermore, EFs set at the higher strength of 400 mV/mm would significantly increase cell migration speed (Figure 2j) and *x*-axis translocation; which describes the distance a cell travels relative to the *x*-axis (Figure 2k). OPCs exposed to a 400 mV/mm EF were found to migrate at a speed of 14.78 ± 1.25 μm/h, which was significantly greater than the speed of OPCs that were not exposed to an EF, which was recorded as an average of 5.62 ± 0.74 μm/h (Figure 2j). After EF stimulation, the OPCs displayed no significant changes to their morphology; which was confirmed by the presence of specific markers such as A2B5 and Olig2 (Figure S2). These results suggest that not only will OPCs respond to external electrical signals to undergo directional migration, but also suggest that OPC migration speed and directedness correlate strongly with the applied EF strength. Approximately 50 cells were analysed in each experiment.

### 2.4. OPC Electrotaxis Is Dependent on β1 Integrin

β1 integrin plays an important part in the activation of downstream signals that regulate cellular mechanisms. The EF-induced upregulation of its subunit has been previously demonstrated in migrating human retinal pigment epithelial cells [24]. Consequently, we endeavoured to examine the link between β1 integrin and the EF-guided migration of OPCs. It was discovered that inhibiting the β1 integrin receptor led to a reduction in OPC directedness (Figure 2i). Cells were observed moving in random directions, even when exposed to a relatively high-strength EF of 400 mV/mm (Figure 2d,h; Supplemental Video 4). Inhibition of the β1 integrin receptor was also found to significantly reduce OPC migration speed and net displacement (Figure 2i–k). When exposed to hamster IgM as a non-specific antibody control, OPC migration patterns did not appear to change (Supplemental Video 5). These results show that the β1 integrin receptor is essential for the directional migration of OPCs.

### 2.5. The Asymmetric Rearrangement of Actin in EF-Guided Migrating OPCs

**Figure 3:**
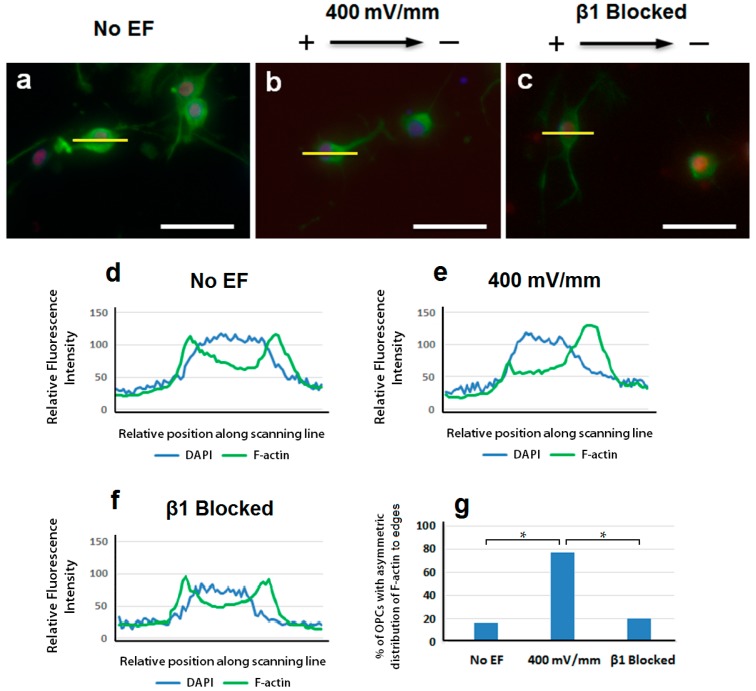
Asymmetric rearrangement of actin in electrotactic OPCs. Without exposure to an EF, there is no obvious asymmetric distribution of actin in OPCs (**a**); Results show an asymmetric reorganization of actin towards the leading edge of rat OPCs following exposure to a 400 mV/mm EF (**b**); Actin localization was observed to be similar in both OPCs that were not EF-treated, and OPCs that were exposed to a 400 mV/mm EF with β1 integrin inhibition (**c**); A fluorescence intensity scan measures the average fluorescence along three lines drawn at random positions in 30 different cells. Linear fluorescence intensity scans (**d**–**f**) were conducted along the yellow lines (shown as examples) in the images (**a**–**c**), respectively. The relative fluorescence intensities generated by ICC-stained DAPI and F-actin were analysed and compared under each experimental condition: no EF (**d**); 400 mV/mm EF (**e**) and 400 mV/mm EF with β1 integrin inhibition (**f**); Quantification results of OPCs with asymmetric rearrangement of F-actin to the leading edge of migrating cells (**g**). Statistical analysis (by χ^2^-test) indicates that the observed differences in asymmetric F-actin distribution between OPCs from the EF group, and OPCs from the groups No EF and EF with β1 inhibition, are both significant (* *p* < 0.05). Composite images (**a**–**c**) are merged images consisting of 3 channels: F-actin (**green**), Olig2 (**red**), and DAPI (**blue**). Scale bar = 50 μm.

Many cellular processes rely on active cytoskeletal dynamics. Cells will employ protrusive leading edges in order to navigate a range of physiological environments. Classical models of leading-edge protrusion depend upon a dendritic actin network that undergoes continuous reassembly in order to form ruffling lamellipodia [25,26]. We have previously demonstrated that specific signaling molecules, such as PIP3 and CLASP2, will tend to accumulate along the leading edges of migrating neural stem/progenitor cells along with various cytoskeletal components—including actin [20,21]. In this study, F-actin was found to be asymmetrically redistributed to favor the leading edges of OPCs migrating in an EF (Figure 3b,e). This redistribution was disrupted in OPCs that were treated with β1 integrin inhibitors (Figure 3c,f). F-actin was found to be asymmetrically redistributed in almost 80% (38 out of 50 cells) of migrating OPCs that were exposed to an EF (Figure 3g). Conversely, only ~20% of OPCs displayed an asymmetric redistribution of F-actin when either not exposed to an EF (7 out of 36 cells), or exposed to a 400 mV/mm EF with β1 integrin inhibition (9 out of 41 cells) (Figure 3g). We also noticed that the low levels of actin rearrangement in non-migrating OPCs were similar across the three conditions mentioned above. Our results imply that the directional migration of OPCs is dependent upon on a link between their cytoskeletal architecture and the external electric field; with the accompanying observations suggesting that this link requires β1 integrin signalling for effective function.

## 3. Discussion

Stem cell-based transplantation therapies have the potential to alleviate serious neurological conditions. Often attending the acquisition of viable cells are relevant investigations into cellular motility and signal-driven migration. Consequently, several methods have utilized either biochemical or biophysical means in an attempt to regulate stem cell migration [21,22,27,28,29]. Electric fields have the capacity to function as guidance cues to regulate the migration of both endogenous and grafted neural stem cells [14,29]. Recent studies, including our own work, have shown that neural stem/progenitor cells (NS/PCs) will respond to electrical stimulation and undergo directional migration [20,21,30,31]. The experiments performed in this study have shown that oligodendrocyte progenitor cells (OPCs) possess a similar capability: the OPCs displayed remarkable electrotaxis when exposed to physiological-strength EFs. These results illustrate the capacity of externally applied EFs to act as guidance cues for OPC migration. Notably, OPC migration speed, directedness and displacement towards the cathode were all found to correlate strongly with EF strength (Figure 2j). Increases in cell migration speed that were dependent on EF strength have been previously demonstrated for both adult rat NPCs (aNPCs) [20] and embryonic brain-derived rat NPCs (eNPCs) [20,30], as well as for rat ventral midbrain-derived dopaminergic NPCs (NPCs^vm^) [21], and even human neural stem cells (hNSCs) [31].

The degree of directedness of rat OPCs appears to be similar to that of rat NPCs^vm^ [21], but is slightly reduced relative to the directedness of migrating rat aNPCs/eNPCs [20]. This roughly places the degree of OPC directedness alongside differentiated cell types such as keratinocytes [32,33] and corneal epithelial cells [34], but above that of human retinal pigment epithelial cells [24] and hippocampal neurons [35]. For several of these cell types, it is proposed that their response to electrical stimulation in vivo serves to facilitate the wound healing process. Damaged tissues have been observed to exhibit endogenous EFs that can be manipulated to enhance cell division and migration [14,32]. Whether the observed in vitro electrotactic response of OPCs will also occur in vivo or not, remains unclear. One study [36], using human embryonic stem cell-derived OPCs (hESC-OPCs) in an animal model of diffuse axonal injury, reported a marked tendency for OPCs to migrate specifically along white matter tracts before maturing into MBP^+^ oligodendrocytes. It could be proposed that this preferential migration occurs as a result of endogenous electrical cues that are triggered by injury, and that the in vitro electrotactic OPC response observed in this study may have an in vivo correlate after all. In a separate study, Li et al. [37] showed that NSC-derived OPCs would undergo cathodal migration and that, despite an increase in cell displacement and directedness, an increased EF strength did not significantly affect OPC migration speed. Hippocampal neurons [35,38], hNSCs [31], NSPCs [30], NPCs^vm^ [21], and both aNPCs and eNPCs [20] have all been observed to undergo directional cathodal migration when exposed to DC EFs set at physiological magnitudes. These reported migratory preferences could well be the result of either cell-specific or species-specific signaling mechanisms that have yet to be explained.

The PI3K/Akt signaling pathway has often been associated with the electrotactic response observed in several types of cells [20]. However, recent studies suggest that different types of neural progenitor cells can respond to electrical stimuli by mobilizing different signaling pathways [21,39,40]. Here, we have demonstrated the importance of the β1 integrin signaling pathway for EF-guided OPC migration, as well as the effects of its inhibition on OPC directedness, migration speed, and displacement. The most notable observation was the significant reduction in OPC directedness when the β1 integrin receptor was blocked by its antibody (Figure 2i). The expression of a particular integrin is dependent on a cell’s developmental stage. Integrins are responsible for relaying various signals between the actin cytoskeleton and the extracellular matrix (ECM) [4], and thus play a role in mediating changes to the cell cytoskeleton. Given the vital role played by the cytoskeleton in cell migration, the marked reduction in OPC directedness as a result of β1 integrin inhibition is somewhat unsurprising. Blocking the β1 integrin subunit has also been shown to significantly decrease oligodendrocyte survival, as well as impair the ability of oligodendrocytes to extend cellular processes in vitro [4]. β1 integrin signaling is crucial for normal oligodendrocyte development, and activates a specific set of proteins that mediate the transmission of signals between various intracellular components and the ECM. In addition to Fyn and focal adhesion kinase (FAK), Akt is one of these proteins, indicating that β1 integrin can influence the canonical PI3K/Akt pathway [4]. Consequently, the PI3K/Akt signaling pathway could play a part in mediating the EF-guided migration of OPCs.

In order to undergo directional migration, cells will become dynamically polarized in response to an extracellular signaling gradient. The polymerization and elongation of actin filaments along the cell’s leading edge will form the protrusions that drive cell movement [37]. Our results show that the leading processes of rat OPCs tend to orient cathodally when exposed to an EF, and hence exhibit a directional preference similar to other types of neural cells [37,38,41]. This has been deduced based upon the appreciable accumulation of actin along the leading edge of the migrating OPCs (Figure 3b). This accumulation has also been observed in eNPCs that were exposed to an EF [20], as well as in cell types outside the nervous system [28,42]. This suggests that if a cytoskeletal rearrangement occurs as a result of EF application, it may occur via a similar mechanism in cells from different lineages. The ARP2/3 complex (actin-related proteins 2 and 3 complex) is one of the regulators of actin nucleation, and hence a regulator of cytoskeletal remodeling [25,26]. The ARP2/3 complex will nucleate new actin filaments that branch from existing filaments, which drives the lamellipodial protrusions necessary for cell migration. It has been demonstrated that actin nucleation by the ARP2/3 complex is essential for NSC-derived OPC migration in an EF; given that ARP2/3^−/−^ OPCs used in one study were observed to migrate in random directions, and at a significantly lower speed than ARP2/3^+/+^ OPCs [37]. Therefore, actin nucleation by the ARP2/3 complex plays a major role in OPC migration, and the directedness of OPCs is dependent upon its function. Given that, in this study, actin was asymmetrically re-distributed towards the leading edge of the migrating OPCs, our results support the existing literature regarding the role played by actin in facilitating OPC directedness.

## 4. Materials and Methods

### 4.1. Isolation, Culture and Differentiation of Rat Oligodendrocyte Progenitor Cells (OPCs)

Ten newborn (≤P2) Sprague-Dawley rat pups were sacrificed using a humane method that conforms to UK government regulations regarding the care and use of laboratory animals (Project approved February 2011 by Cardiff University Animal Welfare and Ethical Review Body (AWERB); Project Identification Code: 30/2816). The method for OPC isolation and culture was performed as described previously [23]. Briefly, the cortices were dissected from each pup and isolated before the meningeal tissues were removed. All meninges-free cortices were transferred into a 7 mL Bijou tube containing 2 mL of cold Dissection Medium (500 mL Dulbecco’s Modified Eagle Medium (DMEM, Sigma, cat. no. D6429, St. Louis, MO, USA) containing 1 mL MycoZap Plus-PR (Lonza, cat. no. VZA-2021, Basel, Switzerland)). The cortices were minced into small pieces prior to papain digestion at 37 °C for 1 h. After spinning down, the supernatant was aspirated and the tissue pellets were re-suspended in 10 mL of Mixed-glial Culture Medium (Dissection Medium supplemented with 10% FBS). The cells were seeded onto poly-d-lysine-coated T75 flasks after the cell suspension was further diluted by 1:10 using Mixed-glial Culture Medium. The culture medium was replaced every 3 days. After 10 days in culture, the OPCs were separated from the mixed-glial cultures using a traditional shaking method, and were cultured thereafter using an OPC Culture Medium (500 mL DMEM containing: 1% 100× SATO supplement; 0.2% MycoZap Plus-PR; 5 μg/mL insulin (Sigma, cat. no. I1882); 50 μg/mL human holo-transferrin (Sigma; cat. no. T0665)); 10 ng/mL PDGF and 10 ng/mL bFGF. Using methods described previously, the OPCs were cultured as oligospheres prior to differentiation into oligodendrocytes. To obtain oligodendrocytes, OPCs were cultured for 5–7 days in OPC Culture Medium supplemented with FBS, T3, and T4, but without growth factors bFGF and PDGF, as described previously [23]. The culture medium was replaced every 3 days.

### 4.2. EF-Directed Cell Migration and Time-Lapse Imaging

OPCs were grown as single cells on poly-d-lysine/laminin-coated electrostatic chambers. Cell migration was assessed using an electrotaxis apparatus, as described previously [43]. Briefly, steady direct current (DC) EFs (in a physiological range of up to 400 mV/mm) were applied through agar-salt bridges connecting silver/silver chloride electrodes in beakers of Steinberg’s solution, to pools of culture medium on either side of the chamber. Before EF application, CO_2_-independent medium (OPC Culture Medium supplemented with 25 mM HEPES) was transferred into the culture chambers. Cells were exposed to an EF for 2–4 h at 37 °C in a temperature-controlled chamber on an inverted microscope stage. Time-lapse images were recorded using a DeltaVision imaging system with a motorized *X*, *Y*, *Z* stage. Cell migration was quantified as described previously [34]. The mean directedness of the total cell population was calculated using the formula $\sum_{i=1}^{n}\cos\theta i/n$, where *n* is the total number of cells and θ*i* is the angle between the vector of cell displacement and the EF vector. Thus, if a cell moves in parallel with the electric field toward the cathode (and hence the target destination), the cosine of the angle will equal 1. If a cell moves perpendicularly to the direction of the electric field, the cosine of the angle will equal 0; and if a cell moves in the direction opposite to that of the applied electric field, the cosine of the angle will equal −1. The net displacement describes the final position of the cell relative to the direction of the applied electric field. Trajectory speed was calculated as the total distance travelled by the cells divided by the travelling time, and describes cell motility. Displacement speed was calculated as the straight line distance between the start and end points of migrating cells divided by the travelling time, which describes the efficiency/persistence of directional migration [21]. To quantify the cell migration speed, directedness, and displacement, cells from three independent experiments were analysed.

### 4.3. OPC β1 Integrin Blocking

In order to inhibit β1 integrin, anti-integrin β1 antibody (10 μg/mL, Hamster Anti-Rat CD29 (Integrin β1 chain) IgM, Clone Ha2/5, BD Biosciences, San Jose, CA, USA) was added to the OPC Culture Medium both 2 h prior to, and during the application of DC EFs in the electrostatic chamber. For the non-specific antibody control, Purified Hamster IgM (Clone G235-1, BD Biosciences) was used [44].

### 4.4. Immunofluorescent Analysis

Following EF-directed OPC migration, or OPC differentiation, the cells were fixed with 4% paraformaldehyde for 20 min, permeabilized with 0.1% TritonX-100 for 10 min, and incubated in blocking solution (5% BSA in PBS) for 30 min prior to incubation with primary antibodies at 4 °C overnight. After extensive washing with PBS, cells were incubated with fluorescence-labelled secondary antibodies at 37 °C for 1 h, washed with PBS, and mounted in Vectashield mounting medium with DAPI (Vector Laboratories, Peterborough, UK). All antibodies were diluted in blocking solution. The primary antibodies used were: anti-Olig2 (1:500, Millipore, cat. no. AB9610, Billerica, MA, USA), anti-A2B5, clone A2B5-105 (1:200, Millipore, cat. no. MAB 312R), anti-NG2 (1:200 Millipore, cat. no. AB5320), anti-MBP (1:400 Millipore, cat. no. 05-675) and anti-F-actin antibody (1:300, Abcam, cat. no. AB205). The secondary antibodies used were: Alexa Fluor^®^ 594 donkey anti-goat IgG and Alexa Fluor^®^ 488 donkey anti-mouse IgG (Molecular probe, Invitrogen, Carlsbad, CA, USA). Nuclei were counterstained with diamidino-2-phenylindole (DAPI). Images were captured using a DeltaVision microscope imaging system (GE Healthcare, Chicago, IL, USA).

### 4.5. Statistical Analysis

Statistical analysis was performed using a two tailed Student’s *t*-test, where the data are expressed as mean ± SD. A value of *p* < 0.05 was considered to be statistically significant. The quantization of images was performed using ImageJ software (National Institutes of Health, Bethesda, MD, USA).

## Supplementary Materials

Supplementary materials can be found at www.mdpi.com/1422-0067/17/11/1948/s1.

## References

[1] Miller G. (2005). Neuroscience. The dark side of glia. Science.

[2] Lappe-Siefke C., Goebbels S., Gravel M., Nicksch E., Lee J., Braun P.E., Griffiths I.R., Nave K.A. (2003). Disruption of Cnp1 uncouples oligodendroglial functions in axonal support and myelination. Nat. Genet..

[3] Richardson W.D., Kessaris N., Pringle N. (2006). Oligodendrocyte wars. Nat. Rev. Neurosci..

[4] O’Meara R.W., Michalski J.P., Kothary R. (2011). Integrin Signaling in Oligodendrocytes and Its Importance in CNS Myelination. J. Signal Transduct..

[5] Tsai H., Niu J., Munji R., Davalos D., Chang J., Zhang H., Tien A., Kuo C.J., Chan J.R., Daneman R. (2016). Oligodendrocyte precursors migrate along vasculature in the developing nervous system. Science.

[6] Zhang S.C. (2001). Defining glial cells during CNS development. Nat. Rev. Neurosci..

[7] Pringle N.P., Mudhar H.S., Collarini E.J., Richardson W.D. (1992). PDGF receptors in the rat CNS: During late neurogenesis, PDGF α-receptor expression appears to be restricted to glial cells of the oligodendrocyte lineage. Development.

[8] Franklin R.J., Ffrench-Constant C. (2008). Remyelination in the CNS: From biology to therapy. Nat. Rev. Neurosci..

[9] Schmidt C., Ohlemeyer C., Labrakakis C., Walter T., Kettenmann H., Schnitzer J. (1997). Analysis of Motile Oligodendrocyte Precursor Cells in Vitro and in Brain Slices. Glia.

[10] Goldman S.A. (2011). Progenitor cell-based treatment of the pediatric myelin disorders. Arch. Neurol..

[11] Cummings B.J., Uchida N., Tamaki S.J., Salazar D.L., Hooshmand M., Summers R., Gage F.H., Anderson A.J. (2005). Human neural stem cells differentiate and promote locomotor recovery in spinal cord-injured mice. Proc. Natl. Acad. Sci. USA.

[12] Franklin R.J., Ffrench-Constant C. (2010). Stem cell treatments and multiple sclerosis. BMJ.

[13] Wu B., Sun L., Li P., Tian M., Luo Y., Ren X. (2012). Transplantation of oligodendrocyte precursor cells improves myelination and promotes functional recovery after spinal cord injury. Injury.

[14] McCaig C.D., Rajnicek A.M., Song B., Zhao M. (2005). Controlling Cell Behaviour Electrically: Current Views and Future Potential. Physiol. Rev..

[15] Nuccitelli R. (2003). Endogenous electric fields in embryos during development, regeneration and wound healing. Radiat. Prot. Dosim..

[16] Borgens R.B. (1988). Stimulation of neuronal regeneration and development by steady electrical fields. Adv. Neurol..

[17] Kessaris N., Fogarty M., Iannarelli P., Grist M., Wegner M., Richardson W.D. (2006). Competing waves of oligodendrocytes in the forebrain and postnatal elimination of an embryonic lineage. Nat. Neurosci..

[18] Paez P.M., Fulton D.J., Spreuer V., Handley V., Campagnoni C.W., Macklin W.B., Colwell C., Campagnoni A.T. (2009). Golli myelin basic proteins regulate oligodendroglial progenitor cell migration through voltage-gated Ca^2+^ influx. J. Neurosci..

[19] Warrington A.E., Barbarese E., Pfeiffer S.E. (1993). Differential myelinogenic capacity of specific developmental stages of the oligodendrocyte lineage upon transplantation into hypomyelinating hosts. J. Neurosci. Res..

[20] Meng X., Arocena M., Penninger J., Gage F.H., Zhao M., Song B. (2011). PI3K mediated electrotaxis of embryonic and adult neural progenitor cells in the presence of growth factors. Exp. Neurol..

[21] Liu J., Zhu B., Zhang G., Wang J., Tian W., Ju G., Wei X., Song B. (2015). Electric signals regulate directional migration of ventral midbrain derived dopaminergic neural progenitor cells via Wnt/GSK3β signaling. Exp. Neurol..

[22] Miyamoto Y., Yamauchi J., Tanoue A. (2008). Cdk5 phosphorylation of WAVE2 regulates oligodendrocyte precursor cell migration through nonreceptor tyrosine kinase Fyn. J. Neurosci..

[23] Zhu B., Zhao C., Young F.I., Franklin R.J., Song B. (2014). Isolation and long-term expansion of functional, myelinating oligodendrocyte progenitor cells from neonatal rat brain. Curr. Protoc. Stem Cell Biol..

[24] Han J., Yan X., Han Q., Li Y., Du Z., Hui Y. (2011). Integrin β1 Subunit Signaling Is Involved in the Directed Migration of Human Retinal Pigment Epithelial Cells following Electric Field Stimulation. Ophthalmic Res..

[25] Pollard T.D., Blanchoin L., Mullins R.D. (2000). Molecular mechanisms controlling actin filament dynamics in nonmuscle cells. Annu. Rev. Biophys. Biomol. Struct..

[26] Insall R.H., Machesky L.M. (2009). Actin dynamics at the leading edge: From simple machinery to complex networks. Dev. Cell.

[27] Zhao Z., Watt C., Karystinou A., Roelofs A.J., McCaig C.C., Gibson I.R. (2011). Directed migration of human bone marrow mesenchymal stem cells in a physiological direct current electric field. Eur. Cell Mater..

[28] Zhao M., Pu J., Forrester J.V., McCaig C.D. (2002). Membrane lipids, EGF receptors, and intracellular signals co-localize and are polarized in epithelial cells moving directionally in a physiological electric field. FASEB J..

[29] Li Y., Wang X., Yao L. (2015). Directional migration and transcriptional analysis of oligodendrocyte precursors subjected to stimulation of electrical signal. Am. J. Physiol. Cell Physiol..

[30] Li L., El-Hayek Y.H., Liu B., Chen Y., Gomez E., Wu X. (2008). Direct-current electrical field guides neuronal stem/progenitor cell migration. Stem Cells.

[31] Feng J.F., Liu J., Zhang X.Z., Zhang L., Jiang J.Y., Nolta J. (2012). Guided migration of neural stem cells derived from human embryonic stem cells by an electric field. Stem Cells.

[32] Zhao M., Song B., Pu J., Wada T., Reid B., Tai G., Wang F., Guo A., Walczysko P., Gu Y. (2006). Electrical signals control wound healing through phosphatidylinositol-3-OH kinase-γ and PTEN. Nature.

[33] Pullar C.E., Baier B.S., Kariya Y., Russell A.J., Horst B.A., Marinkovich M.P., Isseroff R.R. (2006). β4 integrin and epidermal growth factor co-ordinately regulate electric field-mediated directional migration via Rac1. Mol. Biol. Cell.

[34] Zhao M., Agius-Fernandez A., Forrester J.V., McCaig C.D. (1996). Orientation and directed migration of cultured corneal epithelial cells in small electric fields are serum dependent. J. Cell Sci..

[35] Yao L., Shanley L., McCaig C., Zhao M. (2008). Small applied electric fields guide migration of hippocampal neurons. J. Cell. Physiol..

[36] Xu L., Ryu J., Hiel H., Menon A., Aggarwal A., Rha E., Mahairaki V., Cummings B.J., Koliatsos V.E. (2015). Transplantation of human oligodendrocyte progenitor cells in an animal model of diffuse traumatic axonal injury: Survival and differentiation. Stem Cell Res. Ther..

[37] Li Y., Wang P.S., Lucas G., Li R., Yao L. (2015). ARP2/3 complex is required for directional migration of neural stem cell-derived oligodendrocyte precursors in electric fields. Stem Cell Res. Ther..

[38] Yao L., McCaig C., Zhao M. (2009). Electrical Signals Polarize Neuronal Organelles, Direct Neuron Migration, and Orient Cell Division. Hippocampus.

[39] Hotary K.B., Robinson K.B. (1990). Endogenous electrical currents and the resultant voltage gradients in the chick embryo. Dev. Biol..

[40] Hotary K.B., Robinson K.B. (1991). The neural tube of the *Xenopus* embryo maintains a potential difference across itself. Dev. Brain Res..

[41] Li Y., Weiss M., Yao L. (2014). Directed migration of embryonic stem cell-derived neural cells in an applied electric field. Stem Cell Rev. Rep..

[42] Li X., Kolega J. (2002). Effects of direct current electric fields on cell migration and actin filament distribution in bovine vascular endothelial cells. J. Vasc. Res..

[43] Song B., Gu Y., Pu J., Reid B., Zhao Z., Zhao M. (2007). Application of direct current electric fields to cells and tissues in vitro and modulation of wound electric field in vivo. Nat. Protoc..

[44] Tian E., Hoffman M., Ten Hagen K. (2012). *O*-glycosylation modulates integrin and FGF signaling by influencing the secretion of basement membrane components. Nat. Commun..

