# Supplementary material for: Electric Signals Regulate the Directional Migration of Oligodendrocyte Progenitor Cells (OPCs) via β1 Integrin
Source: Int J Mol Sci. 2016 Nov 22;17(11):1948. doi: 10.3390/ijms17111948 (PMC5133942; doi:10.3390/ijms17111948)
Supplement: Supplementary file 1 [file ijms-17-01948-s001.zip › ijms-146912-Supplementary Material/ijms-146912-supplementary material.pdf]

Supplementary Materials: Electric Signals Regulate the Directional Migration of Oligodendrocyte Progenitor Cells (OPCs) via β1 Integrin

Bangfu Zhu, Matthew Nicholls, Yu Gu, Gaofeng Zhang, Chao Zhao, Robin J. M. Franklin and Bing Song

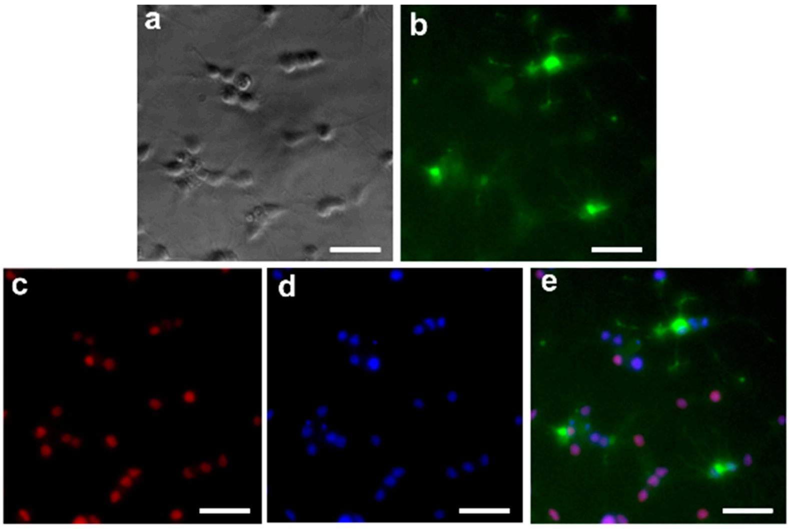

Figure S1. Characterization of differentiating rat OPCs. Differentiating OPCs exhibit a greater number of processes and form secondary branches (**a**). After 5 days under differentiation conditions, some OPCs matured into MBP+ myelin-producing oligodendrocytes (**b**, **green**), while all cells remained Olig2-positive (**c**, **red**). Cell nuclei were counter-stained with DAPI (**d**, **blue**). Merged image of (**b**–**d**) (**e**). Scale bar = 50 μm.

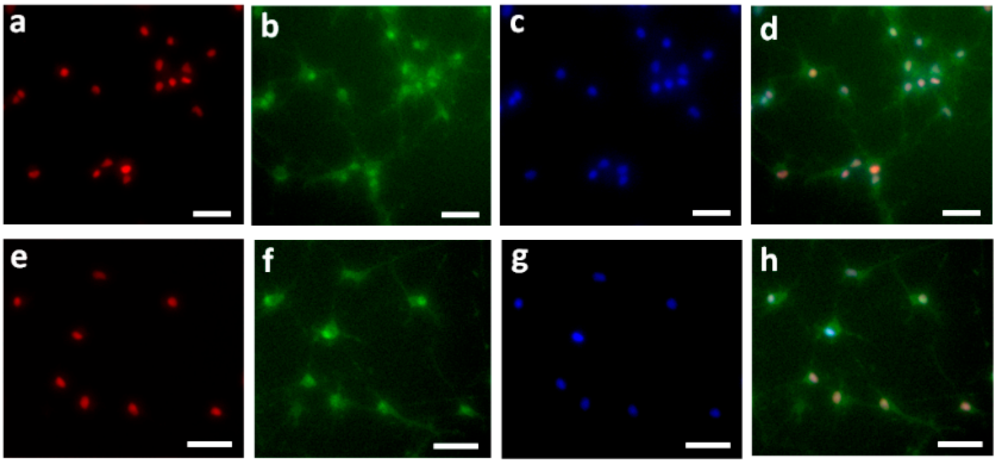

Figure S2. Immunostaining comparison of rat oligodendrocyte progenitor cells (OPCs) before (**a**–**d**) and after (**e**–**h**) EF stimuli. Before EFs, OPCs normally display a branched morphology, which can be characterized using OPC-specific markers such as Olig2 (**a**, **red**) and A2B5 (**b**, **green**). OPCs exhibited no changes in morphology or marker expression following EF stimulation, and remained immunopositive for Olig2 (**e**, **red**) and A2B5 (**f**, **green**). Nuclear counter-staining of OPCs with DAPI (**c**,**g**, **blue**). The DAPI channel is combined with the A2B5 and Olig2 channels (**d**,**h**). Scale bar = 50 μm.
